# High-resolution, on-chip RF photonic signal processor using Brillouin gain shaping and RF interference

**DOI:** 10.1038/s41598-017-06270-4

**Published:** 2017-07-19

**Authors:** Amol Choudhary, Yang Liu, Blair Morrison, Khu Vu, Duk-Yong Choi, Pan Ma, Stephen Madden, David Marpaung, Benjamin J. Eggleton

**Affiliations:** 1 Centre for Ultrahigh bandwidth Devices for Optical Systems (CUDOS), School of Physics, University of Sydney, Sydney, 2006 Australia; 2 Australian Institute for Nanoscale Science and Technology (AINST), University of Sydney, Sydney, 2006 Australia; 3 CUDOS, Laser Physics Centre, Australian National University, Canberra, 0200 Australia

## Abstract

Integrated microwave photonics has strongly emerged as a next-generation technology to address limitations of conventional RF electronics for wireless communications. High-resolution RF signal processing still remains a challenge due to limitations in technology that offer sub-GHz spectral resolution, in particular at high carrier frequencies. In this paper, we present an on-chip high-resolution RF signal processor, capable of providing high-suppression spectral filtering, large phase shifts and ns-scale time delays. This was achieved through tailoring of the Brillouin gain profiles using Stokes and anti-Stokes resonances combined with RF interferometry on a low-loss photonic chip with strong opto-acoustic interactions. Using an optical power of <40 mW, reconfigurable filters with a bandwidth of ~20 MHz and an extinction ratio in excess of 30 dB are synthesized. Through the concept of vector addition of RF signals we demonstrate, almost an order of magnitude amplification in the phase and delay compared to devices purely based upon the slow-light effect of Brillouin scattering. This concept allows for versatile and power-efficient manipulation of the amplitude and phase of RF signals on a photonic chip for applications in wireless communications including software defined radios and beam forming.

## Introduction

In modern radio frequency (RF) communication systems especially for 5G implementations, information is being transmitted in rapidly varying spectral windows at increasingly higher frequencies^1^, requiring fast reconfiguration^2^ and high-resolution RF signal processing^3, 4^ to achieve high spectral efficiencies and agile communication systems. It is challenging for conventional RF electronics to keep up with stringent requirements of wireless communications such as high frequency agility, and reconfigurability. To overcome these challenges, RF photonics^5, 6^ has been developed, simultaneously taking advantage of photonics and RF electronics. A versatile and low power RF signal processor that can perform several functionalities and that can be tailored to the specific requirements of users will reduce complexity and power consumption and be suitable for rapid deployment in sophisticated RF systems.

Stimulated Brillouin scattering^7, 8^ has become very popular for high-resolution and frequency-agile RF photonic signal processing, providing optical filters with unrivalled resolution in the order of megahertz. However, the SBS frequency shift (Ω_B_) and the linewidth (υ_B_) are dependent on the intrinsic material properties and cannot be tuned easily^9^. The amplitude response is also related to the phase response through the Kramers-Kronig relations^10^, therefore this resonance also manifests in a phase response over a very narrow bandwidth. Through SBS in kilometers of fibers, several RF functionalities have been demonstrated including filters^11–16^, phase shifters^17, 18^, delay lines^19, 20^, frequency measurement systems^21, 22^ and beam formers^23^ while SBS filtering has also been used for high-capacity optical communications^24–28^. The replacement of RF electronic components with RF photonic components requires a reduction in the footprint of the devices through integration^29^, along with the reduction in the power consumption and cost. This has motivated the development of on-chip platforms^30, 31^ for generating high SBS gains in silicon^32–34^ and chalcogenides^35, 36^ for various microwave photonic functionalities^37–43^. Nevertheless, in each of these cases the spectral resolution was mainly governed by the material properties, with the increase in the bandwidth possible through a cascade of pumps spaced at different frequencies^39^. In contrast, the reduction in the bandwidth is challenging and could only be achieved by increasing the pump power, negatively affecting the power-efficiency of the device^40^.

An alternative technique to achieve narrow-band optical filters using km-scale fibers, is to sculpt the Brillouin gain (Stokes) response using two Brillouin loss (anti-Stokes) responses at the tail of Lorentzian profile to effectively narrow the optical resonance^44, 45^. Performing this purely in the optical domain to achieve high suppression filters requires high powers increasing the power consumption. The use of high pump powers also adversely affects the linearity of such a filter^46^. To improve the power-efficiency of the filters, it is possible to use RF interferometry to increase the extinction ratio^47^ or to increase the filter slope^48^. The filter response formed with a passband and two narrow stop bands can find applications in shared antenna aperture systems and concurrent transmit–receive systems for isolating dynamic frequencies of interest^49^ as well as for software-defined radio applications^50^.

In this paper, we present advanced RF photonic signal processing through the combination of Brillouin gain-loss tailoring and RF interferometry. By precise control of the individual SBS gain and loss resonances, we achieved a unique high-resolution RF photonic bandpass filtering response with anomalously-high-suppression adjacent stop bands, useful for signal selection in the presence of strong interference. This filter response was also tunable in central frequency up to 40 GHz. We further study the phase response of the filter which reveals that significant enhancement of the phase slope, and subsequently the group delay, can be achieved in the passband. Delay amplification by almost an order of magnitude when compared to a purely SBS slow-light-based delay line was observed, demonstrating low-power pulse delay capability. These findings are consistent with EIT-like phenomena observed in optics, but with critical improvement in performance in terms of passband transmission. The unique combination of the amplitude and delay response of this filter could point to practical implementations of the EIT-analogue concept for high-resolution and power-efficient RF signal processing.

## Results

### Principle of Operation

**Figure 1:**
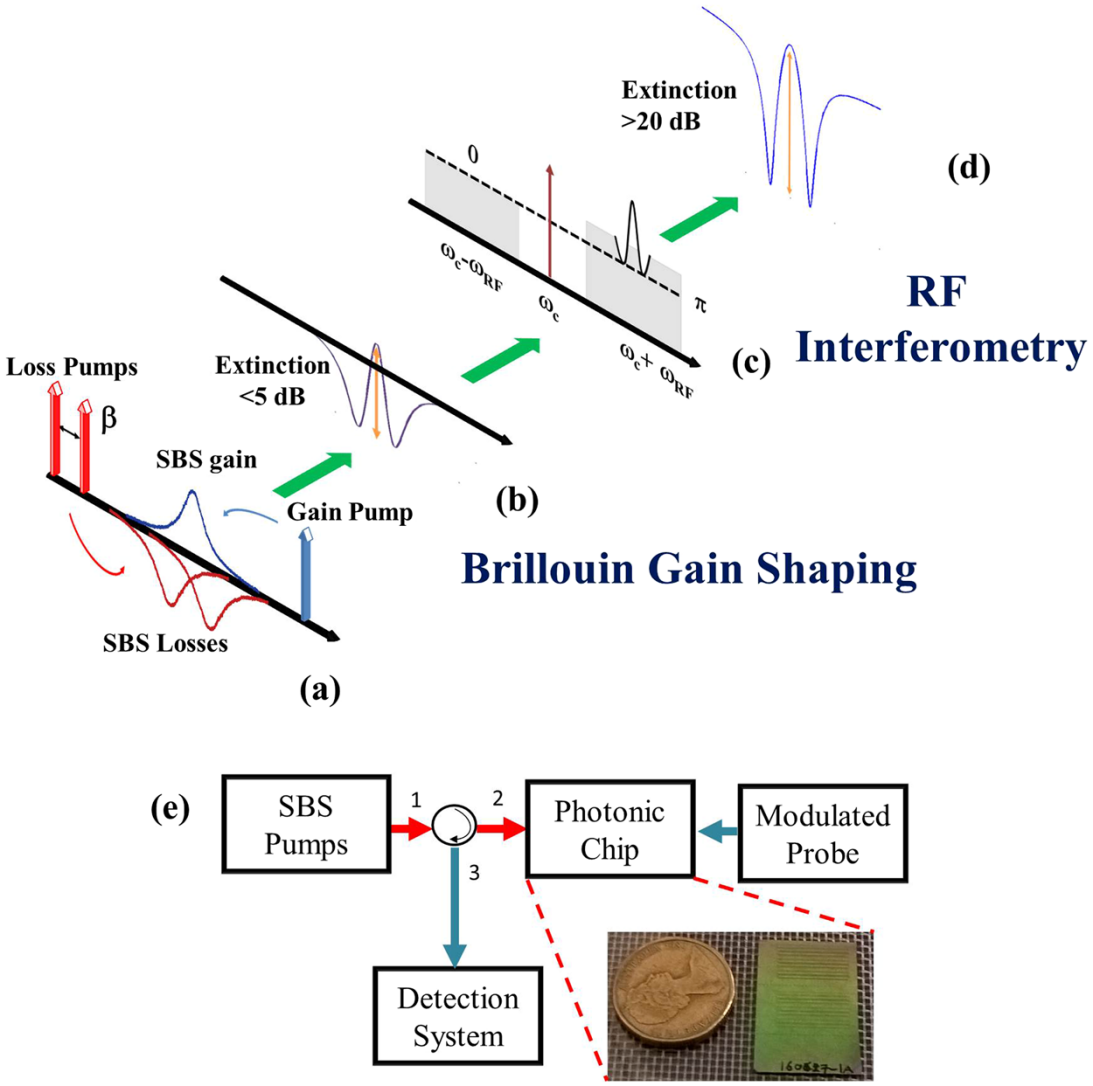
Principle of operation of the RF signal processor in the RF domain. (**a**) One SBS gain pump and two loss pumps were aligned to remove energy from the tail of the Lorentzian profile, (**b**) the optical filter amplitude response, (**c**) the resonance applied to the upper sideband of a phase modulated signal with the lower sideband attenuated, and (**d**) the high suppression filter formed due to RF interference. (**e**) A schematic representation of the experimental setup for realizing an on-chip SBS-based RF signal processor, with a photograph of the photonic chip next to an Australian Dollar coin.

The SBS-based RF photonic signal processor was shaped using a combination of a single SBS gain and two SBS loss resonances^44^ as illustrated in Fig. 1(a). Two SBS loss resonances resulting from the anti-Stokes waves are superimposed at the tail of the SBS gain response, effectively removing energy from the tail of the Lorentzian gain profile. In the amplitude domain, the resulting optical response, measured using single sideband modulation (SSB)^51^ is shown in Fig. 1(b). There is a tradeoff between the bandwidth and the suppression, which are dependent on two degrees of freedom^44^ allowing tailoring of the optical filter profile: i) the separation between the loss pumps which controls the location of the anti-Stokes resonance with respect to the center of the gain profile, and ii) the ratio of the gain pump to the loss pumps which controls the relative power of the Stokes resonance and the anti-Stokes resonances^44, 52^. However, the filter response has a low extinction (as defined in Fig. 1(b)), which depends on the value of the pump powers for gain and the loss. The suppression ratio can be increased by increasing the value of the gain/loss, albeit at the cost of an increased optical power, adversely affecting the power budget of the system. Furthermore, the phase response of this filter has a shallow slope implying a small signal group delay (first derivative of the phase response over the frequency). A delay line made from such a resonance would require large pump powers to achieve modest time delays making such a device power inefficient.

To increase the suppression and the phase slope, we combined the concept of gain tailoring explained above, with the concept of RF interference^16, 40, 53^. RF interference relies on the vector addition of two or more RF signals. Constructive interference may be achieved if the two signals are in phase; alternatively destructive interference is achieved if the phase offset between the two signals is ±π. The vector sum can also result in the change of the phase of the RF signal resulting in phase amplification^54–56^. In practice, we achieved RF interference by encoding the RF signals into the optical domain using a modulation format with arbitrary amplitudes and phase in each sideband (achieved by using a phase modulator and a programmable optical filter) and mixing the processed signals through photodetection. This results in the formation of two sidebands equal in amplitude with a relative phase difference of ±π as seen from Fig. 1(c). At the photodetector, the mixing product between the carrier and two out-of-phase sidebands results in destructive interference, producing only a DC current. On application of the SBS gain to the upper sideband, the condition for destructive interference is not met and a bandpass response is created within the SBS gain bandwidth^57^. On the introduction of the two loss resonances to the edge of the gain profile, and on attenuating the lower sideband to match the lowest level of the loss resonance (see Fig. 1(c)), the condition for destructive interference is met at only two points shown in Fig. 1(c). This places zeroes on both sides of the bandpass RF photonic filter, leading to a narrowband filter response with increased suppression and a profile which resembles the EIT-like resonance^58^ as shown in Fig. 1(d). Similar to an EIT resonance, a corresponding sharp phase slope resulting in an analogous slow-light effect^59^ is also expected in this case. This is due to a vector-sum between two RF signals resulting in phase amplification^54^. The zeroes placed adjacent to the passband can increase the slope at the edges even further. In RF interferometry, amplification of the phase may occur at the edge of a notch filter response^55, 40^; however, in that case, the phase amplification occurs in the stopband thus attenuating the amplitude of the RF signals. In the EIT-like filter response, since the delay amplification occurs in the passband, there is no amplitude penalty and can be very promising for practical applications in microwave delay lines and phase shifters.

### Experimental Setup

An RF photonic link was set up in which an RF signal was up-converted into the optical domain and processed via the on-chip SBS and then down-converted to the RF domain and is described in detail in the methods section. The RF photonic signal processor consisted of four modules as shown in Fig. 1(e): 1) the SBS pumps, 2) the modulated probe, 3) the SBS medium, and 4) the detection system.

#### SBS pumps

One narrow linewidth laser was used to create the SBS gain resonance, and a second narrow linewidth laser frequency downshifted by 2 × Ω_B_ (2 × 7.7 GHz) was used to create the loss resonances. An intensity modulator was used to create two loss responses at the tail of the SBS gain responses through dual sideband-suppressed carrier (DSB-SC) modulation. A low-speed signal generator was driving the modulator to tune the total separation (β) between the two loss responses. The frequency generated by the signal generator (β/2) was varied from 20 MHz to 40 MHz. The pumps were amplified by an Erbium-doped fiber amplifier (EDFA) before being coupled into the photonic chip.

#### Modulated Probe

Another narrow linewidth laser was used as the probe and was modulated by a phase modulator to generate two out-of-phase sidebands. The SBS response created in Fig. 1(b) was used to process the upper sideband shown in Fig. 1(c). The amplitude of the lower sideband was controlled by using a programmable optical filter (which introduced attenuation) to match the SBS loss response on the upper sideband as shown in Fig. 1(c).

#### SBS Medium

A low-loss and high SBS gain photonic chip based on a chalcogenide platform was used as the high-resolution optical processor. A chalcogenide layer (As_2_S_3_) clad with a layer of silica was used for exciting the SBS owing to a high opto-acoustic overlap and high Brillouin gain coefficient^35^. The total propagation length of the waveguide of 24 cm was achieved through arranging the waveguide in a spiral on a chip with a physical length of only 2 cm^36^. A photograph of the chip is shown in Fig. 1(e). The fabrication details are outlined in the methods section. The total insertion loss was 12 dB with a propagation loss of 0.2 dB/cm and a maximum gain >50 dB with a record net gain of 47 dB was achieved using 223 mW of pump power. Such a low-loss and chip with efficient opto-acoustic interactions, enables for low-power pumping to achieve modest gains used for our experiments. The SBS gain characterization of the photonic chip is presented in the supplementary information.

#### Detection system

The signal processed by the photonic chip was detected by a high-speed photodetector, where the RF signal was generated by the beating of the mixed carrier and the sidebands. The amplitude, phase and delay response of the RF signal processor were measured using a vector network analyzer (VNA) which was also driving the phase modulator.

### Filter Results

**Figure 2:**
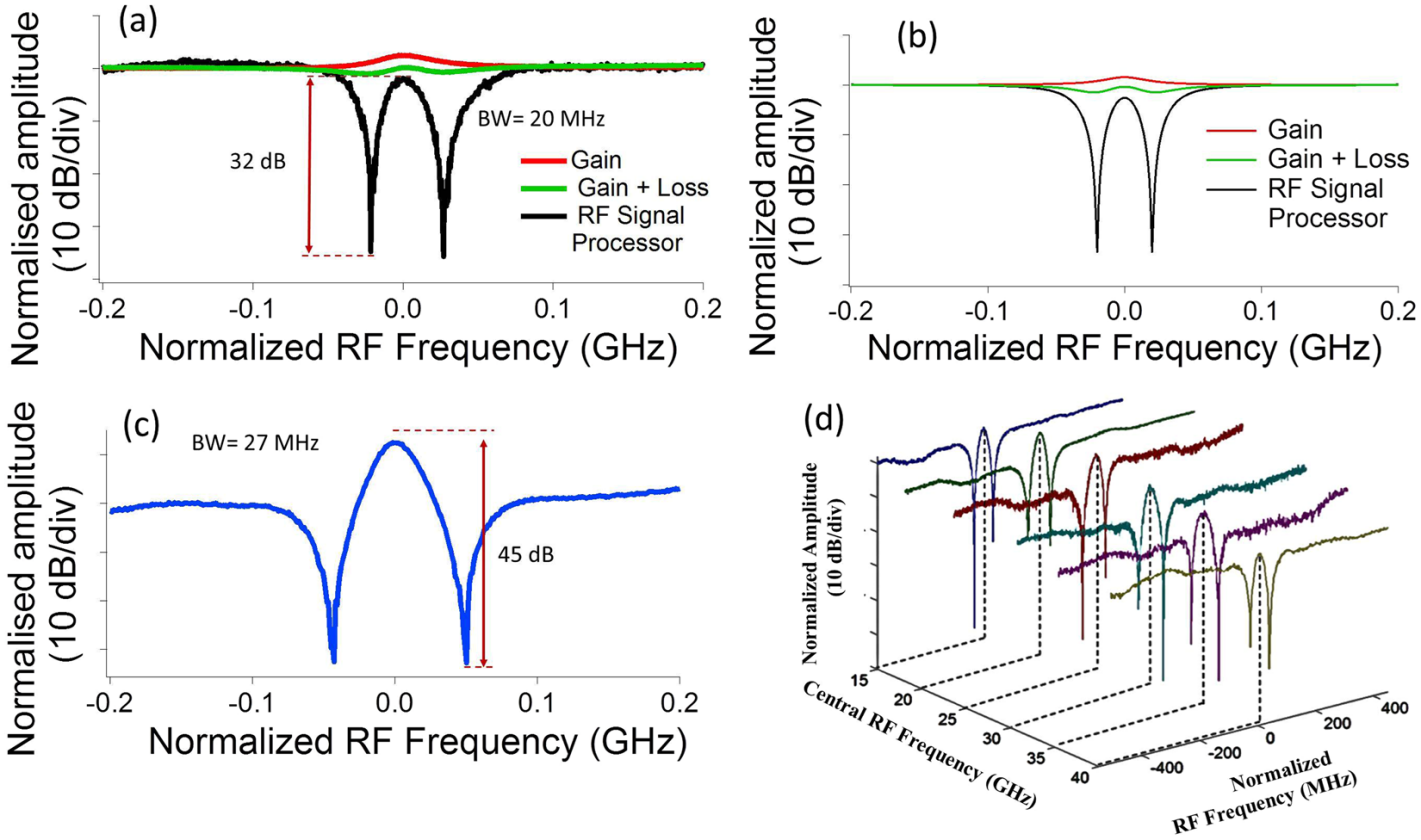
High Resolution filter. (**a**) The SSB response for the gain (red), gain with loss (green) resonance and the RF processor response (black) measured for a signal generator frequency (β/2) of 20 MHz and a total power of 36 mW, (**b**) the simulated results for same experimental conditions, (**c**) a filter formed using β/2 = 25 MHz, total power of 117 mW, and (**d**) the tuning of the central frequency of the filter. The value of β/2 was kept at 30 MHz and the total pump power was kept constant at 36 mW.

The programmable optical filter was set to provide 50 dB attenuation to the lower sideband, thus converting the phase modulation to single sideband (SSB) modulation. The measured SSB trace (S21 parameter on the VNA) for the gain resonance, and the gain with loss resonance is shown in Fig. 2(a). SSB modulation maps the optical response into the RF domain, allowing for high-resolution measurements^51^. The β/2 value was kept at 20 MHz, while the total pump power was kept at 36 mW with the total loss pump power = 22 mW (each loss pump power = 11 mW) and gain pump power = 14 mW. It is observed that the extinction ratio of the filter formed by the SBS gain resonance is only ~1 dB and the bandwidth is 41 MHz. Next, the other sideband was switched ON, by dropping the attenuation of the optical filter to < 1 dB such that the amplitude of the lower sideband matched the amplitudes of the lowest points of the loss responses. This resulted in destructive interference at the lowest points, which resulted in an amplitude response shown in black in Fig.2(a). The measured extinction ratio was 32 dB and the bandwidth was measured to be 20 MHz. Hence this technique reduced the bandwidth by ~2 times, while increasing the suppression by 30 dB, consuming very low optical powers.

The gain response, the gain plus loss response and the RF signal processor response were also simulated and the results are shown in Fig. 2(b). It can be seen that the simulated results have a good agreement with the experimental results. To demonstrate the flexibility of this technique to achieve filters with varying bandwidths and extinction ratios, the separation between the loss pumps and the ratio of the gain power to the loss power was also varied. The values of the β/2 was varied from 20 MHz to 40 MHz for a fixed total power of 36 mW, and then β/2 was fixed at 25 MHz and the total pump power was varied from 36 mW to 117 mW. Different filter configurations for this experiment are shown in the supplementary information. When the separation is increased, the interaction between the Stokes and anti-Stokes waves is reduced, maintaining the original SBS gain’s Lorentzian profile. If for example, the separation between the loss pumps was increased to ~1 GHz, i.e. far beyond the SBS bandwidth, then one gain response and two loss responses will be observed when measured using SSB modulation. The filter profile also depends on the ratios of the SBS gain and loss, since the relative strengths of the gain and loss determines the relative power in the passband and in the tail. Figure 2(a) and (c) represents two filter profiles to demonstrate the tradeoff between the bandwidth and suppression with filter (a) having a suppression of 32 dB and a bandwidth of 20 MHz, and filter (c) having a suppression of 45 dB and bandwidth of 27 MHz. The extinction ratio can be further improved by increasing the frequency separation between the loss pumps and also by increasing the power ratio of the gain to the loss. Both these techniques for tailoring the filter profile can, in principle, be tuned at gigahertz speeds using electro-optic modulators^55^, making these filters of interest for applications in cognitive radios requiring fast reconfiguration^2^.

The frequency agility of our filter was demonstrated by thermally tuning the SBS gain laser and the SBS pump laser to simultaneously shift the gain and the loss response while maintaining the filter shape. The central frequency of the filter was then tuned from 15 GHz up to 40 GHz as shown in Fig. 2(d), with the lower end of the range limited by the frequency roll-off of the programmable optical filter and the higher end being limited by the frequency response of the phase modulator. The use of a Dual Parallel Mach Zehnder Modulator to control the relative amplitude and phase of the two sidebands would not require an optical filter^40^, thus increasing the lower range of the frequency tuning.

### Phase Shifter

**Figure 3:**
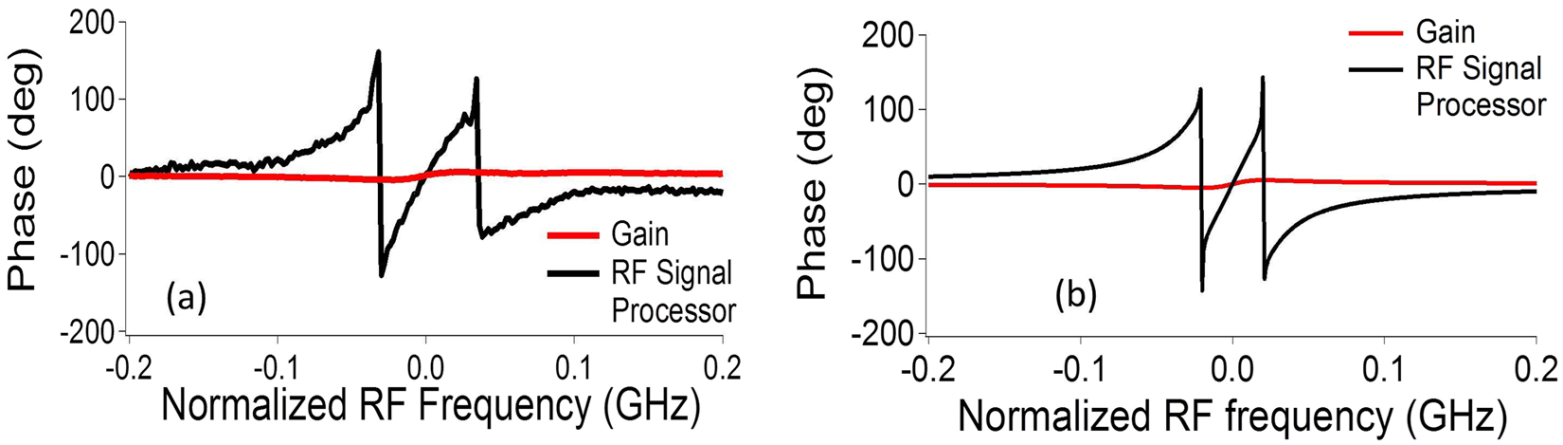
Power-efficient phase shifter. (**a**) The measured S21 phase response of the gain resonance (Red), and the RF processor response (Black). The value of β/2 was kept at 30 MHz and the total pump power was 36 mW, and (**b**) the simulated phase response for the gain (Red) and the RF signal processor (black)

The S21 phase response of the RF photonic signal processor was also measured using the VNA. For low pump powers, the SBS phase response for only the gain resonance is shown (in red) in Fig. 3(a). It can be observed that a total maximum phase swing of only <20° is achieved for the gain resonance. Next, on switching on the lower sideband, RF interference resulted in the introduction of zeroes at the edge of the passband, causing phase amplification by more than an order of magnitude within the passband of the filter. This can be explained through a vector sum addition of two RF signals: the upper sideband and the lower sideband which can be represented by two vectors^54^. In previous approaches, phase amplification has been achieved in a notch filter, where the signal experiences loss^55^. Here, phase amplification occurs within the passband thus reducing the amplitude penalty on the RF signals. A full phase shift of 360° can be achieved by very precisely controlling the amplitude and phase of the sidebands. The simulated results shown in Fig. 3(b) show good agreement with the experimental results. This phase shifter can be of use for applying a large phase shift to a single RF tone over a narrow bandwidth. Of more interest in RF communications is a power-efficient delay line. The first derivative of the phase response gives the delay, which is estimated to be improved by an order of magnitude when compared to an SBS gain resonance. The delay line functionality is discussed in the next sub-section.

### Delay Line

**Figure 4:**
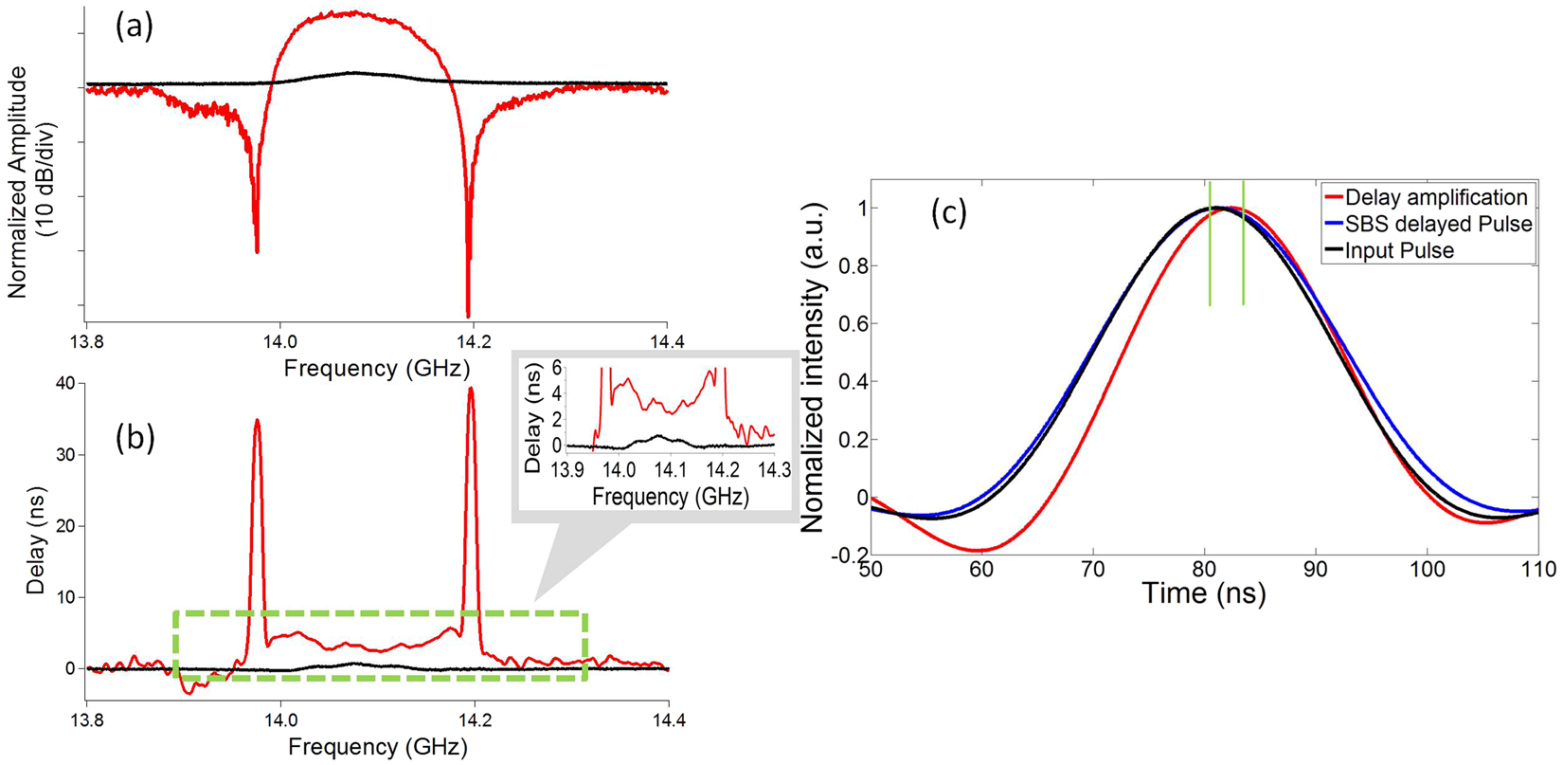
Low power delay line. (**a**) Normalized amplitude response of the 100 MHz filter with a sharp roll-off, and (**b**) the measured delay response. Inset: a zoom of the delay. Black: SSB response for only a gain resonance. Red: RF interference and losses switched ON. (**c**) The pulse delay in the RF signal processor with only the SBS gain resonance (Blue) and with the RF resonance and the loss pumps switched ON (Red) compared to the Input pulse (Black).

The previous implementation of the phase shifter limits the bandwidth to ~20 MHz which is incompatible with pulses <50 ns which are most commonly used in deployed RF systems. A pulse propagated in a narrow delay line would undergo delay amplification but would suffer from severe pulse distortion. In order for delay lines based on this concept to be practical, the bandwidth of the filter was increased to 100 MHz through pump tailoring using an electrical comb modulated onto the SBS pump. The central frequency of the filter was set at 14.1 GHz to fall in the K_u_ band, which is of interest for satellite communications. The synthesized Brillouin response measured using SSB modulation is shown by the black curve in Fig. 4(a). It can be seen that the extinction ratio of the filter is only <3 dB. Switching on the loss pumps and the lower sideband to enable RF interferometry has a two-fold impact: 1) the extinction ratio improves by >40 dB, and 2) the filter becomes more square-shaped due to an improvement in the filter slope. This technique can be of interest in applications that require square-shaped bandpass filters with high extinction ratios. The delay response was measured on the VNA shown in Fig. 4(b) where an order of magnitude amplification in the delay is observed as expected from previous measurements. It is also interesting to note that due to the introduction of the loss pumps at the edge of the passbands, the shape of the delay changes to a concave shape from a convex shape. This technique could thus be useful for introducing chirp to RF pulses.

To verify the delay line, a 23 ns RF pulse was introduced into the bandwidth of the RF signal processor and the output pulse was measured onto an oscilloscope and the results are shown in Fig. 4(c) (the experimental setup is described in the Methods section). The input pulse (black) was delayed by ~0.4 ns by only switching ON the SBS gain pump and switching OFF the lower sidebands as expected from Fig. 4(b). This is the delay expected from a traditional SBS-based delay line for this level of optical pump power. Switching the loss pumps and the RF interference ON increases the pulse delay by almost an order of magnitude as expected from Fig. 4(b) to ~2.4 ns which is in close agreement with the simulated value of 3.3 ns. Thus, this technique allows for the demonstration of delay lines that are much more power-efficient than delay lines based on the slow-light effect of SBS making such devices of great interest in practical RF applications.

## Discussions

In this paper, we have presented an on-chip RF signal processor capable of performing multiple functions including filtering, phase shifting and pulse delay. This was achieved by harnessing the interaction between photons and phonons on a photonic chip through the stimulated Brillouin scattering (SBS) effect. Careful control of Stokes resonances and anti-stokes resonances, enabled the improvement in the resolution of the signal processor, while RF interference amplified the amplitude and phase response of the processor. The filter response contains a narrow passband with two notch responses on each side. This shape resembles the transmission response of electromagnetically induced transparency (EIT) in quantum physics. EIT is a coherent nonlinear process that has a narrow transmission window in a broadband absorption spectrum^58^. EIT has been used in optics owing to the associated sharp change in the refractive index for slow-light applications^59^. EIT has also been extended to find applications in the RF photonics domain^60–62^ for RF signal processing. However, there have been no reports of using the slow-light effect in the RF domain to develop RF delay lines. In this work, besides forming narrowband and high extinction ratio filters, we also take advantage of the sharp phase slope of the EIT-like response to demonstrate phase shifters and delay lines.

Through Brillouin gain shaping and interfering two RF signals, the bandwidth of the filter was reduced by a factor of >2 compared to a filter formed using just the optical response of SBS, using an optical power of only <40 mW. The rejection ratio was also improved by more than 30 dB. We also demonstrate that this filter frequency is continuously tunable up to 40 GHz, marking a major improvement over conventional RF filters. Through further Brillouin gain shaping using multiple pump lines, a broad filter with a sharp slope was also realized. Such filter profiles are of increasing interest for software defined radio and concurrent transmit-receive systems in RF communications. Furthermore, through the strong phase response of this EIT-like RF signal processor, we demonstrate an order of magnitude amplification in the phase shift imparted on an RF signal when compared to a purely SBS-based phase shifter formed using similar pump powers. Finally, a delay line functionality was demonstrated by placing a nanosecond-scale pulse within the bandwidth of the signal processor. The pulse was delayed by almost an order of magnitude more when compared to slow-light effects due to SBS. Such a power-efficient and versatile on-chip RF signal processor capable of performing multiple functionalities, while achieving high resolution and coarse resolution based on optical pump tailoring is of great value in RF communications including beam steering. The current physical size of the device is 2 cm × 1 cm, with modulators and photodetectors outside the chip. In order to meet the demands of integration of RF photonic functionalities, hybrid integration of chalcogenide to achieve large Brillouin gain with Silicon to realize on-chip functional devices is critical. To this end, a first demonstration has recently been made where 18.5 dB of Brillouin gain was achieved in only a 100 μm × 4 mm chalcogenide window opened in a silicon-on-insulator chip^63^. The current implementation requires optical amplifiers to mitigate the insertion loss of the chip which increases the overall power consumption. In the optimized integrated photonic chip, the power consumption will be only dominated by the power consumption of the lasers and will be <2 Watt. Hence using this hybrid technology, we can develop a fully-integrated chip-sized Brillouin RF photonic signal processor in the near future. A value proposition for this processor is that a large delay can be achieved on a chip-scale device at low optical powers, paving the way to demonstrate wideband on-chip true time delay^64^ using SBS^65^.

## Methods

### Photonic Chip fabrication

As_2_S_3_ thin film was deposited via thermal evaporation. Raw As_2_S_3_ material (Amorphous Materials, Garland, Texas, USA) was placed in an electrically heated Tungsten boat in a chamber at low base pressure of 3 × 10^−7^ Torr. The thin film was deposited on <100> oriented 100 mm thermal oxide Silicon wafer. The wafers were mounted on a carousel which was rotated, resulting in a thickness uniformity of less than 1%. The deposition rate was controlled to be constant at 0.1 nm/s. The total film thickness was 930 nm. A ~100 nm SU8 layer was spin-coated on the wafer before the films were annealed in vacuum at 130 °C for 24 h to improve the stability and increase the refractive index from 2.31 to 2.42 at 1550 nm. The waveguide pattern was printed using a 1:1 projection photolithography system. The mask had waveguides with nominal widths of 2.2 µm, 2.4 µm and 2.6 µm, with the 2.4 µm waveguides used in the experiments. The waveguides were arranged in spiral coils with waveguide length in single (2 cm × 1 cm) die of 8.6 cm, 11.7 cm or 23.7 cm. The plasma dry etching was performed in an Oxford Plasmalab 100 ICP RIE system using a mixture gases of CHF_3_, O_2_ and Ar^66^ and an etch depth of 330 nm was targeted. A 630 nm of SiO2 was clad on the waveguides via sputtering for additional acoustic mode confinement. A 10 µm UV-cure polymer layer was then finally coated on top of the wafer. After cleaving the wafers into the chip of the correct dimension, another layer of 270 nm of SiO2 was sputtered to the end facets of the waveguide to act as AR coating and for passivation.

### RF signal processor experimental setup

Teraxion LM lasers with a maximum power of 80 mW and a linewidth of ~100 kHz were used as the gain and loss lasers. The loss laser was modulated by an intensity modulator (10 GHz) driven by a low-speed RF signal generator (100 MHz). The frequencies of the laser were offset by ~15.4 GHz, twice that of the Brillouin shift. The gain laser and the loss laser had a polarization controller to control the polarization of the light coupled to the chip for efficient SBS interactions and were amplified using 1-W EDFAs (Amonics) in each path, after which they were combined using a 50:50 optical coupler. The combined light was sent through a circulator 1, from port 1 to port 2, following which a 1% tap of a 99:1 coupler was used to measure the input pump power. The light was then coupled into the waveguide using lensed fibers with a mode field diameter (MFD) of ~2.5 μm. Another Teraxion LM laser was used as a probe following which a polarization controller, phase modulator (40 GHz) and a programmable filter (Finisar Waveshaper 4000S) were installed to control the modulation format. The probe was then amplified using a low-noise EDFA (Amonics). In principle, one laser can be used as the probe, gain and loss pumps using frequency shifting through the modulation driven by RF tones. The probe laser was then sent through circulator 2 from port 1 to port 2 and then coupled into the other side of the waveguide using 2.5 μm MFD lensed fibers. The probe was then propagated through port 3 of circulator 1 and then sent through a high slope rectangular optical filter (Yenista) to reject the back-reflected pump from the facet of the waveguide. The filtered probe was detected by a photodetector (40 GHz, Finisar) which was connected to port 2 of a 44 GHz vector network Analyzer (VNA) which had windows measuring the S21 amplitude, S21 phase and S21 Delay simultaneously. The output from port 1 was used to drive the phase modulator.

### Pulse delay experimental details

An intensity modulator (IM) was put in the path of the pump and was set to have dual sideband modulation with equalized carrier. 2 RF tones were generated by an RF signal generator (Tabor, 400 MHz) separated by 20 MHz and were modulated onto the IM. The resultant broadened pump was used to increase the bandwidth of the SBS response and thus the filter^14, 39^. 23 ns pulses were generated from an RF signal Generator (Tabor, 1 GHz) and then up-converted from baseband by mixing with a 14.1 GHz RF tone using an RF mixer (Mini Circuits). These pulses were designed to fit within the bandwidth of the filter and were fed into the phase modulator. The RF signal from the photodetector was down-converted to baseband using an RF mixer and measured on a 2 GHz oscilloscope (Rigol). The pulse was measured without SBS gain and was taken as the base trace, and the subsequent delayed pulses were measured for SBS ON and with the RF interference switched ON and the processed plots are presented.

## Electronic supplementary material

Supplementary Information
