# Supplementary material for: High-resolution, on-chip RF photonic signal processor using Brillouin gain shaping and RF interference
Source: Sci Rep. 2017 Jul 19;7:5932. doi: 10.1038/s41598-017-06270-4 (PMC5517533; doi:10.1038/s41598-017-06270-4)
Supplement: Supplementary file 1 — Supplementary Information [file 41598_2017_6270_MOESM1_ESM.doc]

**High-resolution, on-chip RF photonic signal processor using Brillouin gain shaping and RF interference**

**Amol Choudhary^1,2^*, Yang Liu^1.2^, Blair Morrison^1,2^, Khu Vu^3^, Duk-Yong Choi^3^, Pan Ma^3^, Stephen Madden^3^, David Marpaung^1,2^, and Benjamin J. Eggleton^1,2^**

1 Centre for Ultrahigh bandwidth Devices for Optical Systems (CUDOS), School of Physics, University of Sydney, Sydney, 2006, Australia

2 Australian Institute for Nanoscale Science and Technology (AINST), University of Sydney, Sydney, 2006, Australia

3 CUDOS, Laser Physics Center, Australian National University, Canberra, 0200, Australia

**Supplementary Information**

**SBS gain characterization.** The Brillouin gain was characterized using a pump-probe setup [1] using 2 narrow linewidth lasers. The peak gain vs. on-chip power is shown in Figure S1 (a) and the measured SBS gain spectrum for maximum pump power of 223 mW is shown in Figure S1 (b). It can be seen that a maximum gain of 52 dB is achieved. The total insertion loss was measured to be 12 dB, out of which 4.8 dB was attributed to the propagation losses and 3.6 dB/facet was attributed to the coupling losses. The net gain was therefore calculated to be 47 dB, which is the highest ever reported on-chip SBS gain.


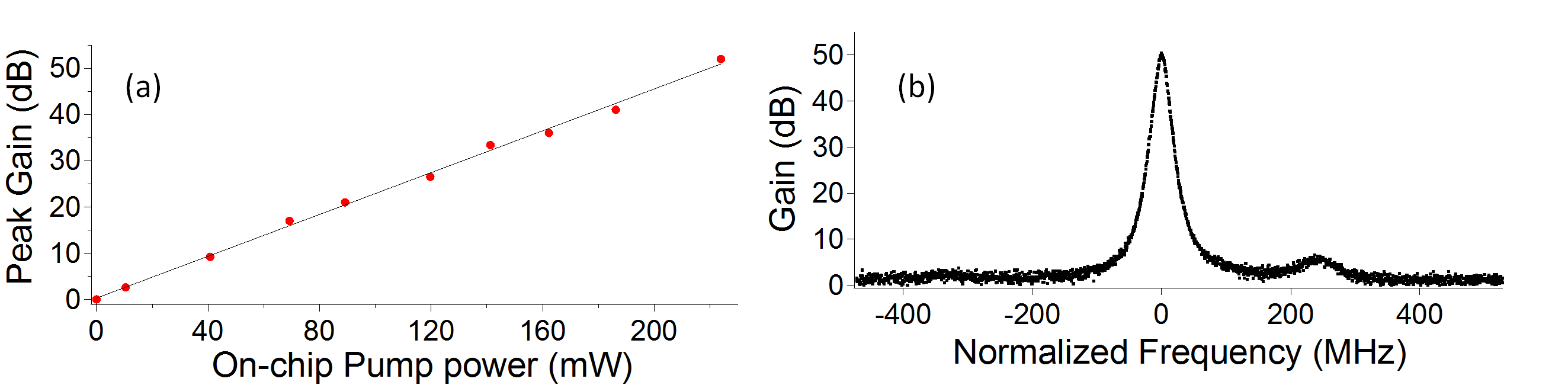


**Figure S1.** (a) The peak SBS gain vs. on-chip pump power for the chalcogenide waveguide, and (b) the SBS gain spectrum for a pump power of 223 mW and a peak SBS gain of 52 dB.

**Tailoring of the Filter responses.** The filter profiles were tailored by changing the separation between the loss pumps β and filters formed with different conditions for values of β/2 of 20 MHz, 25 MHz, 30 MHz and 35 MHz for a fixed total power of 36 mW and are shown in Figure S2. The slight offset in the central frequencies is due to the relative drift of the pump and the probe lasers. This can be mitigated by frequency locking the lasers.


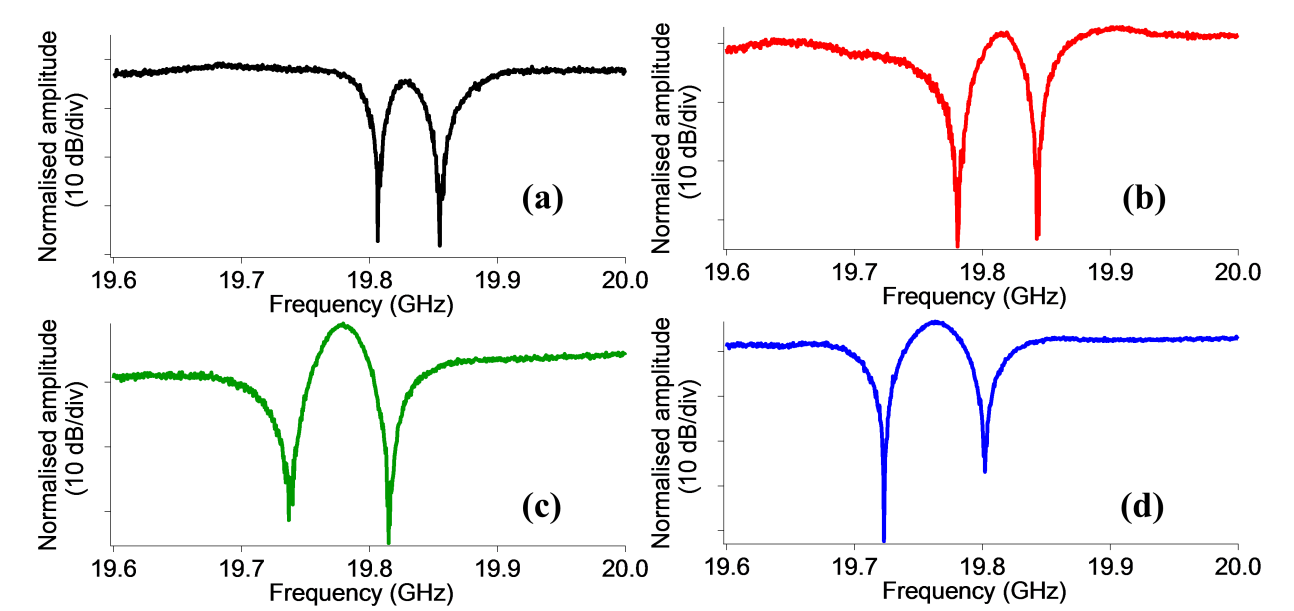


**Figure S2.** The filter responses formed by controlling the separation between the SBS losses using a total coupled pump power of 36 mW. β/2= (a) 20 MHz, (b) 25 MHz, (c) 30 MHz, and (d) 35 MHz.

The filter profiles were then tailored by controlling the ratio of the gain pump power to the loss pump power for a fixed β/2 of 25 MHz. The total power was changed from 36 mW to 117 mW as shown in Figure S3.


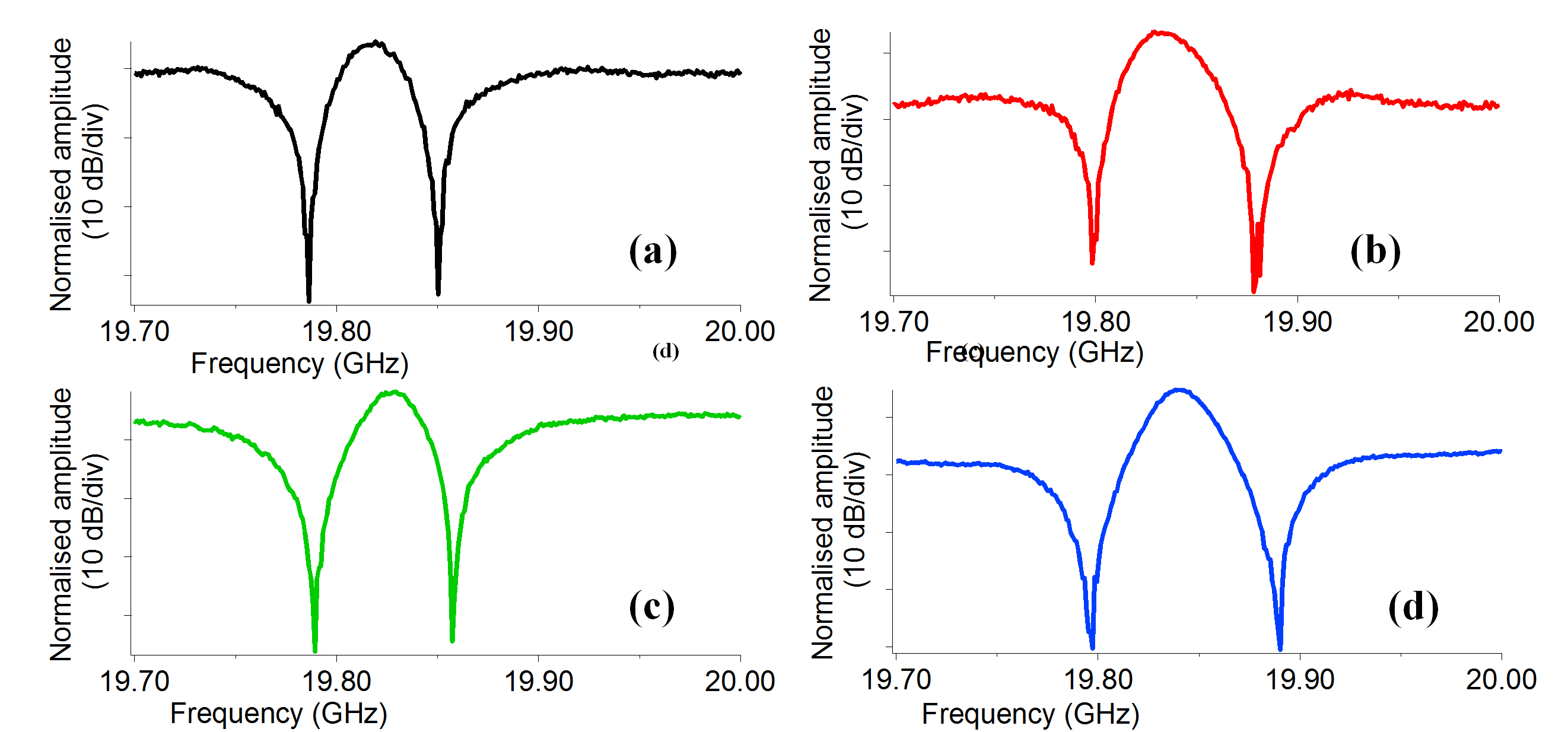


**Figure S3.** The filter responses formed by using different combinations of the gain power (G), and the loss power (L) for β/2 = 25 MHz. The total power was varied: (a) 36 mW with G= 14 mW and L= 22 mW, b) 49 mw with G= 23 mW and L= 26 mW, c) 73 mW with G= 28 mW and L= 45 mW, and d) 117 mW with G= 42 mW and L=75 mW.

**References**

[1] A. Loayssa, R. Hernández, D. Benito, and S. Galech, Characterization of stimulated Brillouin scattering spectra by use of optical single-sideband modulation, *Opt. Lett.* 29, 638-640 (2004)
